# Intra-Tumoral Genomic Heterogeneity in Rectal Cancer: Mutational Status Is Dependent on Preoperative Biopsy Depth and Location

**DOI:** 10.3390/cancers13092271

**Published:** 2021-05-09

**Authors:** Floris A. Vuijk, Carlijn van de Water, Shannon Lent-van Vliet, Maxime J. M. van der Valk, Femke Simmer, Cornelis J. H. van de Velde, Alexander L. Vahrmeijer, Iris D. Nagtegaal, Denise E. Hilling

**Affiliations:** 1 Department of Surgery, Leiden University Medical Center, 2333 ZA Leiden, The Netherlands; f.a.vuijk@lumc.nl (F.A.V.); m.j.m.van_der_valk@lumc.nl (M.J.M.v.d.V.); vdwater.c@gmail.com (C.J.H.v.d.V.); a.l.vahrmeijer@lumc.nl (A.L.V.); 2 Department of Pathology, Radboud University Medical Center, 6525 GA Nijmegen, The Netherlands; Carlijn.vandeWater1@radboudumc.nl (C.v.d.W.); Shannon.vanVliet@radboudumc.nl (S.L.-v.V.); femke.doubrava-simmer@radboudumc.nl (F.S.); iris.nagtegaal@radboudumc.nl (I.D.N.); 3 Department of Surgical Oncology and Gastrointestinal Surgery, Erasmus MC Cancer Institute, University Medical Center Rotterdam, 3015 GD Rotterdam, The Netherlands

**Keywords:** rectal cancer, intra-tumor heterogeneity, next generation sequencing

## Abstract

**Simple Summary:**

A subset of patients with rectal cancer are treated before surgery with chemoradiation. Unfortunately, this neoadjuvant chemoradiotherapy does not have the preferred effect of tumor downstaging in all patients, but does bring substantial side effects and possible complications. A pre-treatment prediction based on available parameters might provide a means to better select therapy for individual patients. Genomic mutational status of pre-treatment biopsies may provide prognostic information, however, it also might be influenced by tumor heterogeneity. This study investigates whether pre-treatment biopsy material is a reliable way of defining mutational status in rectal cancer.

**Abstract:**

Neoadjuvant therapy before surgical resection is indicated for patients with locally advanced rectal cancer. However, a significant number of patients show minimal or no response to neoadjuvant therapy. Unfortunately, we are currently unable to predict response and identify non-responding patients before neoadjuvant treatment is given. Genomic mutational status might provide valuable prognostic information. However, it is unclear whether predictions based on genomic mutational status in single preoperative biopsies are reliable due to intra-tumoral heterogeneity. In this study we aim to investigate the reliability of genomic mutations found in single pre-operative biopsies by comparing genomic mutations to four other locations within the same tumor using next generation sequencing. Rectal cancer patients undergoing primary resection without neoadjuvant therapy were included. From each patient, one biopsy, two deep and two superficial samples were obtained and sequenced using a targeted next generation sequencing gene panel. Concordance between these five samples was assessed. In this feasibility study we included 11 patients. In 7 out of 11 (64%) patients, all 5 samples showed concordant mutations. In 4 out of 11 patients (36%) discordant mutations were observed. In conclusion, assessment of mutational status on a single pre-operative biopsy shows discordance with tumor tissue from other locations in 36% of cases. These results warrant careful interpretation of biopsy material analysis, as these might be influenced by tumor heterogeneity.

## 1. Introduction

Locally advanced rectal cancer (LARC) patients are currently treated with neoadjuvant (chemo)radiotherapy followed by surgical resection [1]. In clinical practice, the observed response to neoadjuvant therapy is heterogeneous. A pathological complete response (complete regression of tumor and/or pathological lymph nodes) is seen in 15–20% of patients; whereas in the vast majority of patients (54–75%), neoadjuvant therapy results in a partial response [2,3]. Unfortunately, a subset of 10–50% of LARC patients receives futile neoadjuvant treatment when minimal or no response is observed [2,4]. Currently, treatment stratification and prognosis is based on clinical TNM stage, tumor distance to the mesorectal fascia and the presence of extramural vascular invasion [5]. Response prediction based on parameters readily available before neoadjuvant treatment might provide a means to ensure patient-tailored treatment and reduce unnecessary waiting periods and therapy-related toxicity in non-responders. 

Tumor associated immune response and intra-tumoral heterogeneity might be involved in causing therapeutic resistance of the tumor to neoadjuvant therapy [6]. Intra-tumoral genomic heterogeneity refers to the presence of genetically distinct subclones within cancer lesions, and is developed by tumors in reaction to a diversity of microenvironmental factors, including hypoxia, tissue stiffness, immune response and chronic inflammation or can be caused by the polyclonal origin of these tumors [7,8]. Intra-tumoral genomic heterogeneity is particularly significant in colorectal cancer, and is attributed to the presence of both microsatellite- and chromosomal instability [9,10,11]. 

In previous studies, the value of several clinical, pathological and radiological parameters in predicting response to neoadjuvant therapy has been assessed [12,13,14,15,16,17,18,19,20]. Unfortunately, these studies have not resulted in clinically used prediction models so far. The predictive value of genomic mutations in colorectal cancer has previously been investigated; the studies concluded that *KRAS*, as well as *RAS*, *BRAF* and *PIK3CA* mutations, are predictive of tumor response to anti-EGFR therapy [17,18,21,22,23,24,25]. Furthermore, a high degree of intra-tumoral genomic heterogeneity has been associated with worse disease-free survival and was correlated with a higher rate of liver metastases [26]. So far, no specific genomic mutations have been found to accurately predict response to neoadjuvant therapy in LARC patients [19]. 

A combination of genomic mutations might provide valuable prognostic information. However, the reliability of next generation sequencing performed on routinely obtained single preoperative biopsies has yet to be established. Intra-tumoral heterogeneity has been shown to be significant in rectal tumors and their associated lymph nodes and metastases [27,28]. Therefore, genomic mutations found in single preoperative biopsies might vary within individual patients, depending on the biopsy location and depth.

In this study we aim to investigate the reliability of genomic mutations found in a single preoperative biopsy by comparing these mutations to four other locations within the same tumor using next generation sequencing for genes frequently mutated in colorectal cancer.

## 2. Materials and Methods

### 2.1. Patients

Rectal cancer patients from the Radboud University Medical Center, Nijmegen, the Netherlands, and diagnosed between 2010 and 2012 with a biopsy-confirmed rectal adenocarcinoma, were retrospectively included in this study. To exclude any influence of neoadjuvant therapy on the results, only patients undergoing direct surgical resection of the primary tumor (without neoadjuvant chemo- and/or radiotherapy) were included. 

Patient characteristics were obtained from medical records, including age, gender, clinical and pathological characteristics. This project was conducted in accordance with the Declaration of Helsinki and did not require approval of the local IRB according to local WMO regulations.

### 2.2. Tumor Identification and DNA Isolation 

From each patient, five tissue samples were obtained from representative formalin-fixed paraffin-embedded (FFPE) tumor blocks containing material of 1 preoperative diagnostic biopsy, 2 superficial tumor tissue samples and 2 deep (central) tumor tissue samples of the resected specimen. Optimal FFPE blocks (with adequate tumor cellularity of ≥20% from full samples, and >10% in biopsy samples) for smMIP analysis were identified and marked by an expert pathologist (I.N.) on representative hematoxylin and eosin (H&E) stained slides. To obtain sufficient genomic DNA, marked tumor areas were cut out from 10 sequential (non-stained) slides (each 6 µm thick). DNA was isolated at 56 °C for 1 h using TET-lysis buffer with 5% Chelex-100 (Bio-Rad, Hercules, CA, USA) and 400µg proteinase K (Qiagen, Valencia, CA, USA), followed by inactivation at 95 °C during 10 min [29]. The DNA concentration was determined using the Qubit High Sensitivity Kit (Invitrogen, Carlsbad, CA, USA) per manufacturer’s protocol. 

### 2.3. SmMIP Sequencing 

A panel of 911 smMIPs was used to detect variants in 31 cancer-related genes, as displayed in Table 1. To provide gender control, smMIPs targeting AMELX and AMELY were included. The smMIP sequencing protocol has previously been clinically validated and used in the Radboud University Medical Center [29]. One hundred nanogram of isolated DNA was included per sample. After sample preparation, manual library preparation was performed [29]. The purified libraries were diluted. Sequencing was performed using the NextSeq500 (Illumina, San Diego, CA, USA) per manufacturer’s protocol (300 cycles High Output sequencing Kit, Illumina, San Diego, CA, USA), resulting in 2 × 150 bp paired-end reads. 

### 2.4. Sequence Data Analysis 

**Figure 1:**
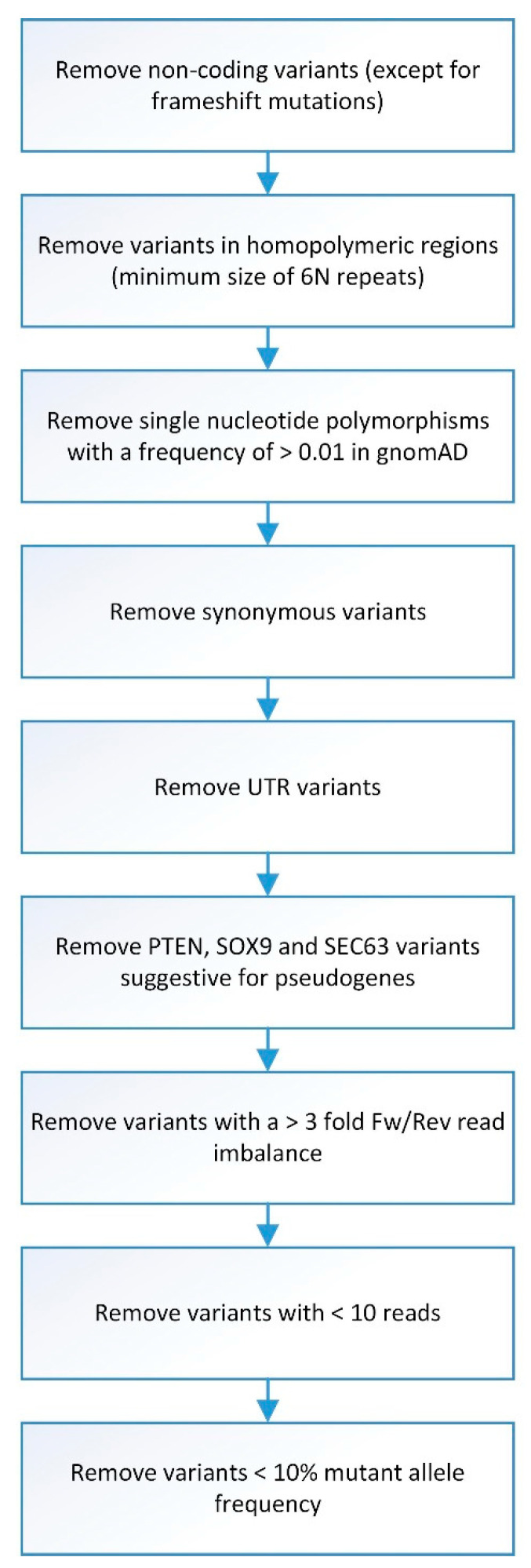
Flowchart of smMIP analysis data filtering. Overview of steps involved in data filtering before smMIP data analysis was performed.

Sequence data were generated from the NextSeq500, after which Bcl to FASTQ conversion and demultiplexing of barcoded reads were automatically performed. Sequence Pilot software (JSI Medical Systems GmbH, Ettenheim, Germany) was used for generating consensus reads and variant identification, with settings as previously described [29]. Variants found in samples passing gender control and exceeding an average minimum reading depth of 180 were automatically filtered with an in-house Python script, as depicted in Figure 1. This threshold excludes, with a certainty of >95%, the presence of a mutation at minimally 10% mutant allele frequency within covered regions. As SOX9 and SEC63 have many pseudogenes resulting in uncertainty regarding found mutations, we have excluded these from further analysis. Due to a technical sequencing artifact (in all samples), PTEN mutation c.407G>A was excluded from the analysis. 

### 2.5. Statistical Analysis 

Statistical analysis was performed using SPSS version 23 (SPSS, Inc., Chicago, IL, USA). Numerical data is presented as mean (standard deviation) or median (interquartile range) based on distribution. Categorical data is presented as frequencies and percentages. In order to quantify tumor heterogeneity, differences in mutational status between biopsies, deep and superficial tumor samples were analyzed by calculating the percentages of concordance and discordance. Concordance was defined as all five samples (1 biopsy, 2 deep samples, and 2 superficial samples) showing identical (or no) mutations. Discordance was defined as ≥1 mutation(s) in any of the 5 samples, which was not found in any of the other samples. For all tests performed, *p* < 0.05 was considered statistically significant. 

## 3. Results

### 3.1. Patients

Data and tissue of 11 patients were included in this study. Patients were on average 72 ± 27.4 years old and included six males and five females. Of these, nine had a pT3 tumor and two a pT4 tumor. All patients were diagnosed with a UICC stage 2 or 3 tumor (Table 2). All patients were treated with immediate resection of the rectal tumor, without prior chemo- and/or radiotherapy. The rectal tumor was on average located 57.8 ± 46.3 mm from the anal verge and measured 53.5 ± 21.6 mm in diameter. Patient 7 had a poorly differentiated tumor (UICC grade 3), whereas all the other patients had a moderately/well differentiated tumor (UICC grade 1–2). All tumors were microsatellite stable. Detailed clinicopathological features are summarized in Table 2. 

### 3.2. Mutation Concordance

**Figure 2:**
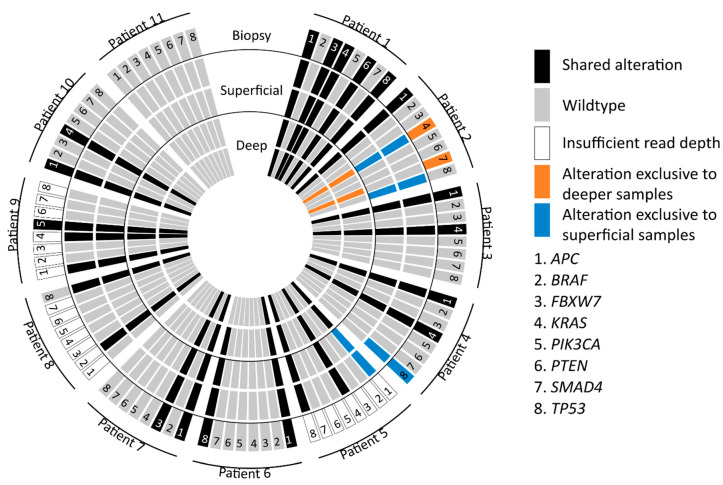
Graphical display of mutations in all samples. Representation of *APC*, *BRAF*, *FBXW7*, *KRAS*, *PIK3CA*, *PTEN*, *SMAD4*, *TP53* mutations found in deep, superficial and biopsy samples. The blue, orange and black colors represent the location of found mutations and possible relation to specifically the deep, superficial or biopsy specimen.

**Figure 3:**
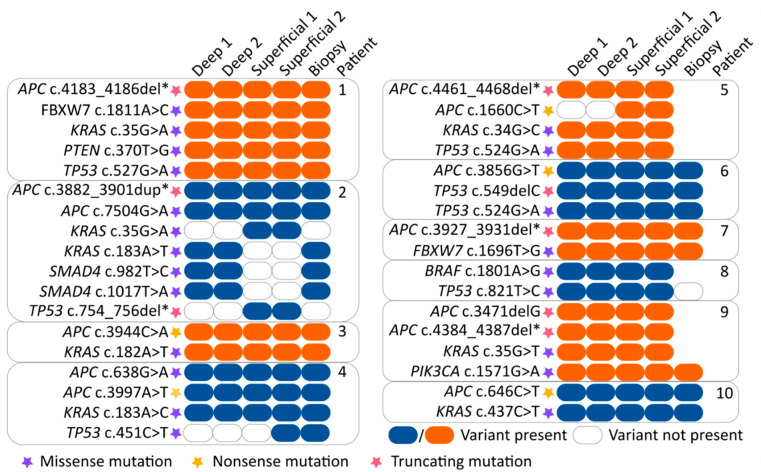
Overview of specific mutations found in all samples. The various mutations are represented by orange and blue colored boxes (blue/orange box = mutation variant is present and clear box = mutation variant is absent). Purple, yellow and red stars indicate the function of the found mutation (purple = missense mutation, yellow = nonsense mutation and red = truncating mutation). The location of the tumor sample is indicated at the top of the boxes. Abbreviation: del*, deletion of several nucleotides. dup*, duplication of several nucleotides.

Twenty-eight genomic mutations were found in the following eight genes: *APC* (9/11), *BRAF* (1/11), *FBXW7* (2/11), *KRAS* (7/11), *PIK3CA* (1/11), *PTEN* (1/11), *SMAD4* (1/11) and *TP53* (6/11). Insufficient (partial) read depth was found in biopsy samples of three patients (patient 5, 8 and 9). In 7 out of 11 (64%) patients, all 5 samples showed concordant mutations. In 4 out of 11 patients (36%) a discordance in mutations was observed within the 5 samples. In patient 2, a discordance in *KRAS* (2 different mutations), *SMAD4* and *TP53* mutations was found between the superficial sample and the biopsy, as well as, both deep samples. Patient 4 showed discordance as the *TP53* mutation was only found in the biopsy and one of two superficial samples. Patient 5 showed discordance for one of the two *APC* mutations. This APC mutation was only found in the superficial samples compared to the deep samples (biopsy results were not available). In patient 8, discordance was found as different *TP53* mutations were found in the biopsy compared to the deep and superficial samples. These results are depicted in Figure 2 and Figure 3. 

Interestingly, patients 4, 5 and 8 have one discordant mutation, whereas patient 2 has five. No differences in differentiation grade, microsatellite status, or tumor stage were found to explain this difference. However, patient 2 was the only patient with a mucinous tumor at pathological examination, whereas all the other patients had not otherwise specified adenocarcinomas. 

In this study, 13 *APC* mutations were found, of which 11 most likely result in loss of function (5 non-sense and 6 frameshift mutations). Regarding *TP53* mutations, 5 missense mutations have been found which are non-functional according to the TP53-IACR database [30]. Furthermore, the effect of the other two *TP53* mutations (one frameshift and one frame deletion) is unclear. All but one *KRAS* mutations are activating hotspot mutations, and the *BRAF* mutation was found in very close proximity to the real hotspot and most likely also results in increased *BRAF* activity [31,32,33]. When compared to previous results from the TCGA study in rectal cancers, the percentage of found mutation frequencies is similar [34]. 

When putting these found mutations into a clinical perspective, only *KRAS* mutations are currently of primary influence in colorectal cancer patients, as these are predictive for cetuximab and panitumumab therapy success. Interestingly, two *KRAS* hotspot mutations (*KRAS* c.35G > A and *KRAS* c.183A > T) were discordant. 

## 4. Discussion

Response to neoadjuvant therapy is heterogeneous in LARC patients [2,4]. Adequate stratification based on parameters available before treatment might enable better use of neoadjuvant therapy. In this light, genomic mutational status might provide valuable prognostic information.

In this study, genomic mutations in pre-operative biopsies were compared to four other locations within the same tumor using next generations sequencing. In 36% of the patients, evaluation of genomic mutational status on a single pre-operative biopsy has shown discordance between the various tumor samples. This illustrates the genomic variability in rectal cancer and could explain the difficulties experienced so far in obtaining reliable biomarkers. These results are in line with previous evidence supporting the presence of intra-tumoral genomic heterogeneity in a considerable proportion of rectal cancers [35]. Three previous studies have compared genomic mutations in up to three intratumoral locations. Hardiman et al. reported up to 10 coding variants uniquely corresponding to 1 of 3 of the tumor locations in their study of 6 patients [35]. In the study of Bettoni et al., only 27% of the observed mutations corresponded to all three samples of a single rectal adenocarcinoma in one patient [36]. On the other hand, Dijkstra et al. reported no differences in mutational status between deep and superficial colorectal cancer tissue in 30 patients [37]. However, the spatial distance between compared tumor samples in this study was limited, as samples were taken from serial sectioning of FFPE blocks three times every 1.2 mm. This might have resulted in serial sectioning of one tumor clone (and thus no difference in found mutations), whereas our study (and others) used tumor samples with a higher spatial distance recovered from various tumor locations.

This study has several limitations. First of all, the small sample size. Moreover, insufficient read depth was achieved in biopsy material from three patients. Therefore, we could not identify variants at all target regions for these samples. Also, the limited targeted next generation sequencing panel might have influenced the interpretation of our results. The number of discordant cases might actually be higher, as this targeted gene panel only provides information on a selected number of mutations. Furthermore, the tumor cell percentage in several samples was low, which may have resulted in mutant allele frequencies below the calling threshold. Lastly, there is no 100% certainty the found mutations were not germ-line mutations, however, considering the observed allelic frequency, this is very unlikely. 

To increase the reliability of the biopsy analysis, the use of multiple and possibly even deeper/larger preoperative biopsies might provide a better representation of intra-tumoral heterogeneity. However, this might also increase the risk of procedure-related complications. A second possibility might be the application of whole exome sequencing or larger targeted gene panels (such as the TSO500, Illumina, San Diego, CA, USA), as this possibly provides a more elaborate analysis of genomic mutations, as compared to next generation sequencing using a limited targeted gene panel. Using these techniques, the mutant-allele heterogeneity (MATH) score was developed to quantitatively assess the spread of allele frequencies and has been correlated to response [19,38]. However, as sampling errors are innate to the biopsy technique, parameters derived from full tumor imaging might be preferable to incorporate characteristics of all genetic subclones present in these cancers. Following this, predicting algorithms should therefore include various clinical, radiological and pathological parameters to overcome the complexity of tumor heterogeneity. 

## 5. Conclusions

In conclusion, assessment of mutational status on a single pre-operative biopsy shows discordance with tumor tissue from other locations in 36% of cases. These results warrant careful interpretation of biopsy material analysis, as they might be influenced by tumor heterogeneity. 

## Figures and Tables

**Table 1:** Overview of regions targeted by Transcan smMIP panel.

Gene	Transcript ID (RefSeq)	Transcript ID (Ensembl)	Exon Number	Targeted Regions	Positions Analyzed for Variants
*ACVR1B*	ENST00000257963	NM_004302	02	Activin types I and II receptor domain	c.92−5 to c.331+5
			03–09	Transforming growth factor beta type I GS-motif	c.556 to c.1518+5
*ACVR2A*	ENST00000241416	NM_001616	06–11	Protein kinase domain	c.673−5 to c.1542+5
*AMER1*	ENST00000330258	NM_152424	02	WTX Protein	c.639 to c.1629
*APC*	ENST00000257430	NM_000038	01–16	Whole gene	c1−5 to c.8532+5
*ARID1A*	ENST00000324856	NM_006015	11–12	ARID DNA-binding domain	c.2989 to c.3397
			20	SWI/SNF-like complex subunit BAF250/Osa	c.5820 to c.6777
*B2M*	ENST00000558401	NM_004048	02	Immunoglobulin C1-set domain	c.68−5 to c.346+5
*BRAF*	ENST00000288602	NM_004333	15	Codon D594-K601	c.1742−5 to c.1860+5
*CASP5*	ENST00000393141	NM_004347	02–03	CARD domain	c.8 to c.433+5
*CASP8*	ENST00000358485	NM_001080125	07–09	Caspase domain	c.838 to c.1617+5
*CTNNB1*	ENST00000349496	NM_001904	03	Codon D32-S45	c.36 to c.163
			08	Codon W383-N387	c.1082−5 to c.1185+5
*EGFR*	ENST00000275493	NM_005228	12	Receptor L domain	c.1391 to c.1498+5
			18–21	Protein tyrosine kinase	c.2062−5 to c.2625+5
*ERBB2*	ENST00000269571	NM_004448	18–24	Protein tyrosine kinase	c.2101 to c.2970+5
*FBXW7*	ENST00000281708	NM_033632	07–12	WD domain, G-beta repeat	c.1035 to c.2124+5
*GNAS*	ENST00000371085	NM_000516	08–09	Codon R201 and Q227	c.586−5 to c.718+5
*IDH2*	ENST00000330062	NM_002168	04	Codon R140 and R172	c.374−5 to c.534+5
*KRAS*	ENST00000311936	NM_004985	02	Codon G12, and G13	c.1−5 to c.111+5
			03	Codon A59 and Q61	c.112−5 to c.232
			04	Codon K117 and A146	c.291−5 to c.385 and c.402 to c.450+5
*MET*	ENST00000318493	NM_001127500	15–21	Protein tyrosine kinase	c.3140 to c.4227+5
*NRAS*	ENST00000369535	NM_002524	02	Codon G12 and G13	c.1−5 to c.99
			03	Codon A59 and Q61	c.135 to c.272
*PIK3CA*	ENST00000263967	NM_006218	10	Codon E542 to Q546	c.1557 to c.1664+5
			21	Codon M1043 to G1049	c.3041 to c.3207+5
*POLE*	ENST00000320574	NM_006231	03–13	DNA-directed DNA polymerase, family B, exonuclease domain	c.205−5 to c.1301
*PTEN*	ENST00000371953	NM_000314	05–08	Dual specificity phosphatase, catalytic domain, C2 domain of PTEN tumor-suppressor protein	c.310 to c.1026+5
*RNF43*	ENST00000407977	NM_017763	02–10	Whole CDS	c.1−5 to c.2352+5
*SMAD2*	ENST00000262160	NM_005901	02–11	Whole CDS	c.1−5 to c.1404+5
*SMAD4*	ENST00000342988	NM_005359	03–04	MH1 domain	c.250−5 to c.454+5
			09–12	MH2 domain	c.956−5 to c.1659+5
*SMARCA2*	ENST00000349721	NM_003070	15–21	SNF2-related, N-terminal domain	c.2185−5 to c.3078+5
			23–25	Helicase, C-terminal	c.3136 to c.3684+5
*SMARCA4*	ENST00000450717	NM_001128846	15–21	SNF2-related, N-terminal domain	c.2275−5 to c.3168+5
			23–25	Helicase, C-terminal	c.3324 to c.3374+5
*SMARCB1*	ENST00000263121	NM_003073	05–09	SNF5/SMARCB1/INI1	c.501−5 to c.1158+5
*SOX9*	ENST00000245479	NM_000346	01–03	Whole CDS	c.1−5 to c.1530+5
*TCF7L2*	ENST00000369397	NM_030756	01–06	CTNNB1 binding, N-terminal	c.1−5 to c.719+5
			09–10	High mobility group box domain	c.933−5 to c.1200+5
*TGFBR2*	ENST00000359013	NM_001024847	04	Codon E125	c.339−5 to c.529+5
*TP53*	ENST00000269305	NM_000546	03–08	P53 DNA-binding domain	c.83 to c.919+5

**Table 2:** Patient characteristics.

Variables		*N* = 11
Age (years)	Mean (SD)	72.2 (27.4)
Gender	Male	6 (55%)
	Female	5 (45%)
pT	3	9 (82%)
	4	2 (18%)
pN	0	6 (55%)
	1	3 (27%)
UICC stage	2 2A 3A 3C	2 (18%) 6 4 1
EMVI Differentiation (UICC grade)	Yes	4 (36%)
	No	6 (55%)
	Missing	1 (9%)
	Well/moderate (UICC grade 1–2)	9 (82%)
	Poor (UICC grade 3)	1 (9%)
	Missing	1 (9%)
Distance to CRM (mm)	Mean (SD)	14.1 (7.7)
Diameter tumor (mm)	Mean (SD)	53.5 (21.6)
Total number of lymph nodes	Median (IQR)	15 (12–19)
Number of tumor positive lymph nodes	Median (IQR)	0 (0–3)
Distance from anal verge (mm)	Mean (SD)	57.8 (46.3)

Abbreviations: UICC grade, Union for International Cancer Control pathological differentiation grade; SD, standard deviation; pT, clinical tumor stage; pN, clinical nodal stage; EMVI, extramural vascular invasion; CRM, circumferential resection margin; IQR, interquartile range.

## Data Availability

The datasets generated and analyzed in this study are not publicly available for the reason of protecting patients’ privacy but are available from the corresponding authors on reasonable request.
